# Brain Resilience and Its Association with Post‐Stroke Dementia: A Neuroimaging‐Based Study

**DOI:** 10.1002/alz70856_101113

**Published:** 2025-12-24

**Authors:** Changi Kim, Mi‐Young Oh

**Affiliations:** ^1^ Seoul national university, Seoul, Seoul, Korea, Republic of (South); ^2^ Bucheon Sejong medical hospital, Bucheon‐si, Gyeonggi‐do, Korea, Republic of (South)

## Abstract

**Background:**

Brain age, a neurobiological construct derived from structural neuroimaging, reflects overall brain health and holds promise for various clinical applications. Brain resilience, defined as the difference between brain age and chronological age, represents an individual's ability to maintain brain health relative to their age. Lower brain resilience may indicate increased vulnerability to neurodegenerative conditions, while higher resilience suggests better brain health. However, the role of brain age and resilience in post‐stroke dementia has not been extensively studied.

**Method:**

Data were collected from 81 patients admitted to Bucheon Sejong Hospital between October 2016 and May 2022. Brain age was derived using FreeSurfer software, which segments T1‐weighted MRI to extract 153 features related to cortical thickness, surface area, and subcortical volume (available at https://github.com/freesurfer/freesurfer). Of these, 77 key features were used to predict brain age using a ridge regression model trained on a healthy cohort aged 18–75 years (available at https://www.photon‐ai.com/enigma_brainage). Brain resilience was defined as the difference between brain age and chronological age.

**Result:**

Of the 81 patients, 58 were diagnosed with post‐stroke dementia. Brain resilience was significantly associated with post‐stroke dementia (OR: 1.07, 95% CI: 1.01–1.13, *p* = 0.02). Additionally, higher national institutes of health stroke scale scores were associated with a higher risk of dementia (OR: 1.16, 95% CI: 1.01–1.13, *p* = 0.02).

**Conclusion:**

Our findings suggest that brain resilience, as measured by the difference between brain age and chronological age, may be a critical factor in the development of post‐stroke dementia. These results indicate that brain age and resilience could serve as valuable biomarkers for predicting post‐stroke dementia and guiding clinical interventions.

